# Does a Species’ Extinction–Proneness Predict Its Contribution to Nestedness? A Test Using a Sunbird-Tree Visitation Network

**DOI:** 10.1371/journal.pone.0170223

**Published:** 2017-01-19

**Authors:** Charles A. Nsor, Hazel M. Chapman, William Godsoe

**Affiliations:** 1 School of Biological Sciences, University of Canterbury, Christchurch, New Zealand; 2 Department of Biological Sciences, Gombe State University, Gombe, Nigeria; 3 Biological Sciences Department, Lincoln University, Lincoln, New Zealand; University of Northampton, UNITED KINGDOM

## Abstract

Animal pollinators and the plants they pollinate depend on networks of mutualistic partnerships and more broadly on the stability of such networks. Based mainly on insect-plant visitation networks, theory predicts that species that are most prone to extinction contribute the most to nestedness, however empirical tests are rare. We used a sunbird-tree visitation network within which were both extinction prone vs non extinction prone sunbird species to test the idea. We predicted that the extinction prone species would contribute the most to nestedness. Using local abundance as a proxy for extinction risk we considered that locally rare sunbird species, by virtue of their small population size and associated demographic stochasticity to be more at risk of extinction than the common species. Our network was not strongly nested and all sunbird species made similar contributions to nestedness, so that in our empirical test, extinction proneness did not predict contribution to nestedness. The consequences of this finding remain unclear. It may be that network theory based on plant-insect mutualisms is not widely applicable and does not work for tree- sunbird mutualistic networks. Alternatively it may be that our network was too small to provide results with any statistical power. Without doubt our study highlights the problems faced when testing network theory in the field; a plethora of ecological considerations can variously impact on results.

## Introduction

Pollinator declines are of global concern for biodiversity and food security [1] so that understanding how plant-pollinator networks respond to perturbations has applications in both conservation biology and agricultural science [2]. For example, individual species make different functional contributions to network properties, so that their decline will have differing implications for the stability of a network [3, 4]. This is especially relevant in networks where one/some of the players are threatened with extinction [3]. In some cases losing a single species will trigger cascade effects, where the loss of one species sets off a cascade of secondary extinctions [5]. However, it is difficult to use current network theory to accurately predict how the loss of single species within a network affects the stability of the whole network; there is controversy in the literature as to which network properties most influence species survival and network stability [6, 7, 8].

Two network properties, nestedness-a tendency for species with fewer partners to interact with a subset of species that interact with more partners or generalist species [9] and connectance—the number of observed links between plant and animal species in a given network divided by the total number of potential links in the network [10, 11]-are among the few network properties where the differential contribution of individual species can be readily evaluated. They are relatively easily incorporated into conservation monitoring and show potential in conservation management [12]. Most mutualistic networks are nested to some degree; nestedness reduces competition relative to facilitation [3], so that the more nested the network, the more resilient it may be to environmental perturbations and species extinctions [3, 13]. Increasing connectance may also increase network resilience because it increases the proportion of all possible interactions that actually occur [11, 14], so that if one species is lost other connections are still available. Theoretically, the removal of strong contributors to either nestedness or connectance negatively affects network stability [3, 13]. One prediction from topological models with direct relevance to conservation is that strong contributors to nestedness are most prone to extinction [3].

The implications of species loss to network stability have mostly been investigated in terms of species network properties or place in network architecture, ignoring their biological characteristics such as temporal availability [11] and pollination efficiency [15, 16]. Moreover most predictions are based on models using empirical insect-plant visitation data sets e.g. [3, 13, 17, 18, 19, 20]. By necessity such models are limited in that they fail to account for relevant biological properties which are understandably difficult to quantify. Even the conservation status of most insects is unknown, which leads to difficulty when including extinction proneness in models.

Bird-plant pollination networks offer good examples of networks where individual species contributors are in decline. Specialised pollinating systems such as e.g., nectar feeding hummingbirds in the New World [21, 22, 23] and sunbirds (Nectarinididae) in the Old World [24, 25, 26] are especially vulnerable. Sunbirds, the focus of this study, are extremely sensitive to habitat loss [25, 26] and at least 15 of 122 sunbird species are either vulnerable (4 species), near threatened (8 species) or endangered (3 species) and are now of global conservation concern. We hoped that some of the limitations inherent in insect-plant pollination networks could be overcome by using bird-pollination networks; birds are physically larger than insects, they have been studied for a long time and their species distributions are well known. Of significance is the fact that although very few pollinating bird species exist, a high proportion of them are of conservation concern [27].

Here we use an empirical sunbird-tree pollination network to test hypotheses around individual species contributions to networks. This is important, especially when network theory is being integrated into conservation management tools [28]. Our aim is to determine how generally applicable are models based on insect-plant visitation data to other animal-plant pollination networks. Never to our knowledge, have these predictions been tested in a visitation network, especially one involving birds exclusively.

Our three objectives were to:

1. Determine the stability of the sunbird-tree visitation network
2. Test if extinction prone species contribute the most to nestedness
3. Test if strong contributors to nestedness have low connectance.

Our network is not closed in that sunbirds visit herbs as well as trees [29, 30] and the trees in our network are occasionally visited by other bird species [31]. At least one tree species (*Anthanotha noldea*) in our network is totally dependent on sunbirds for seed set [32]. We ranked the contributing sunbird species in terms of rarity. On the premise that i) strong contributors to nestedness are the most prone to extinction [3] and that ii) rare species are more extinction prone than common ones [4] we empirically test the hypothesis that rare species contribute the most to nestedness. If our results support the hypothesis, we will better understand biologically why, in this plant-visitation network, strong contributors to nestedness are most prone to extinction. We also investigate the relationship between species contribution to nestedness and their connectance. We hypothesise that strong contributors to nestedness will have low connectance and therefore be more vulnerable to partner species extinctions.

That sunbirds have a known distribution and conservation status allowed us to test our system based on proxy IUCN criteria. This would be much more difficult, if not impossible, with insect taxa, where there are often gaps in our knowledge as to the extinction risk of many species. Moreover, our study is the first time to our knowledge that species rarity has been used as a biological characteristic to test the theoretical implication of species contribution to nestedness. Previous studies have mostly focused on abundance without consideration to rarity and other biological attributes such as size and dietary preference [33, 34].

## Materials and Methods

**Fig 1:**
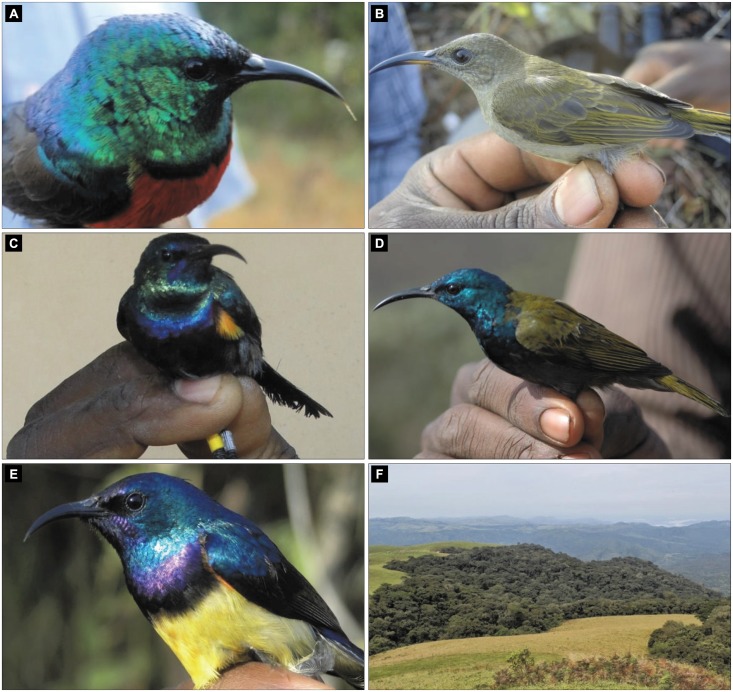
Adult males of some of the common sunbird species in our study area and typical sunbird habitat. A. Northern double collared sunbird, B. Olive sunbird, C. Orange tufted sunbird, D. Green headed sunbird, E. Variable sunbird and F. Ngel Nyaki forest edge.

Our study was conducted in and around Ngel Nyaki Forest Reserve (NNFR; 07^°^ 05' N 11^°^ 04' E) on the eastern edge of the Mambilla Plateau in Taraba State, Nigeria at an elevation of approximately 1650 m a.s.l. NNFR is 46 km^2^, comprising *c*. 7.5 km^2^ of montane forest surrounded by overgrazed *Sporobilus* grassland and small riparian forest fragments [35] (Fig 1). Sunbirds use a variety of habitats in NNFR, including forest, forest edge and riparian fragments [36] and can be envisaged as an island of sunbird habitat within a sea of grassland. Ngel Nyaki forest is the only forest of its kind remaining on the Mambilla Plateau [35].

Interaction data were obtained by direct observation, we identified five individual trees of each of 14 tree species between 2011–2014 and observed all sunbird species visiting them. The tree species were chosen based on flower availability, with the aim being to collect data from as many flowering tree species in the forest as was logistically feasible. Of the 14 tree species, two are IUCN Red Data Listed (*Dombeya ledermanii* and *Nuxia congesta*) but both are locally common [35]. To account for variation in the spatial abundance and distribution of sunbird species, we selected focal trees of each species from different parts of the forest. Trees of each species were always at least 100 metres apart. Observations were restricted to times of the day when sunbirds are particularly active (6:30 am-12:30 pm and 3:30–5:30 pm) and to periods of peak flowering (October-January). Each individual tree was monitored for a period of 20 minutes during each observation session (i.e., morning or evening), for a total of 40 observation minutes per tree per day. A total of 120 minutes were spent observing each tree (i.e. three observation days per tree per species). We alternated the order that tree species were observed during each session to account for any possible biases from time of day.

During observations, we positioned ourselves approximately 25 metres from the focal tree to avoid distracting the sunbirds from foraging. Each time birds were observed visiting a tree, we recorded the number and identity of birds, the time of detection or arrival, the number of flowers contacted, and the time of departure. We used the total number of individuals of each sunbird species that contacted flowers to calculate the frequency of visitation for each tree species. These data were used to develop the flower visitation network.

A line transect census technique [37] was used to estimate the diversity and abundance of sunbirds across the study site. Sampling was carried out between 6:30 am-9:30 am and 3:30–5:30 pm on most days between November 2012-February 2013 and between November 2013- February 2014. A total of 19 transects ranging from 336 m to 1,737 m long and covering a total distance of 16.7 kilometres were surveyed. Transects were selected to include a representative sample of all habitat types in NNFR, which we reasoned would provide a valid representation of the diversity, abundance and distribution of sunbird species in the area. Thirteen transects were located along the edges of the core forest on the eastern side of the reserve, while the other six were scattered within riparian fragments. Each transect was surveyed twice each season (November 2012-February 2013 and November 2013-February 2014), for a total of four sessions per transect. All transects were sampled equally so that both the forest and its surrounding fragment were equally represented. Since sunbirds generally occupy the same niche and utilize similar resources [38, 39], we expected all species of sunbirds to have equal probability of being observed.

During each observation session, the observer and an assistant walked slowly along each transect, recording birds seen within 50 m on either side of the transect. A pair of Nikon 9 x 40 binoculars was used to confirm the identity of birds. Only birds visually identified were included in the census data as it was difficult to estimate the actual number of individuals based on calls alone.

No specific permits were required for the described field studies. Bird observations were from a distance and did not involve endangered or protected species. Bird identification was based on Borrow and Demey (2001) [39].

To determine if strong contributors to network stability are the most prone to extinction we first ranked the sunbird species in our network in order of abundance, our proxy for extinction proneness (Table 1). Our rationale was based on the reasoning of Vidal et al. [4] who used rarity as a proxy for extinction proneness based on IUCN assessment criteria [4, 26]; rare species by virtue of their low population size are more vulnerable to extinction than abundant species [4]. In our case we were working at the local level and based our estimates on local relative abundance rather than IUCN assessment criteria. While all of the sunbird species in our network are IUCN least concern, they vary markedly in their abundance at NNFR. For example two very common residents of NNFR, the Northern Double Collared Sunbird (*Cinnyris reichenowi)* and the orange tufted sunbird (*Cinnyris bouvieri*) are Birdlife International range restricted, while in contrast the Olive sunbird (*Cyanomitra olivaceus)* is extremely uncommon at Ngel Nyaki, yet in Nigeria is classified as a common resident [39]. Using local abundance as a proxy for rarity in our network seemed sensible as the Mambilla Plateau is an isolated massife, surrounded by extremely degraded grassland habitat which presumably limits sunbird dispersal.

**Table 1:** The abundance of sunbird species in the network based on number of observations from the transect survey. Based on IUCN assessment criteria rare species (those with small population sizes) are more extinction prone than common ones.

Sunbird	Species	No.	Rank Abund.	D.D.	Con.	C.N.
V	*Cinnyris venustrus*	525	1	12	0.857	1.2231945
Ndc	*Cinnyris reichenowi*	359	2	12	0.857	0.9510778
Ot	*Cinnyris bouvieri*	356	3	11	0.785	1.1386220
Gh	*Cyanomitra verticalis*	97	4	7	0.5	0.9500930
Cop	*Cinnyris cupreus*	28	5	2	0.142	0.6979206
Sp	*Cinnyris coccinigastrus*	20	6	2	0.142	0.7045720
Coll	*Hedydipna collaris*	0	7	5	0.357	-0.7298658

Ndc-*Northern double-collared sunbird*, *V-Variable sunbird*, *Ot-Orange-tufted sunbird*, *Gh-Green—headed sunbird*, *Cop-Copper-headed sunbird*, *Sp-Splendid sunbird*, *Coll-Collared sunbird; No*.*–number observed during transect survey; Rank Abund*.*- Rank abundance*, *1 most abundant; E*. *Risk- Extinction Risk; D*.*D*.*- Degree distribution;Con*.- *Connectance; C*.*N*.- *Contribution to nestedness*.

To build the tree-sunbird visitation network we combined the observed flower visits (cumulative visits by individuals of each sunbird species) to create a single visitor by plant (*S* x *T*) matrix, or visitation network, in which the cell values represent the number of occasions individual sunbird visitors from species *S* were observed foraging on flowers of tree species *T* [40]. To calculate the nestedness of the whole network (N_tot_) we used the bipartite package and the metric NODF (Nestedness based on Overlap and Decreasing Fill) [41], in R statistical software version 2.15.3 [42]. Using the “visweb” function, we generated a bipartite matrix to visualise the frequency and pattern of interactions in the network. To determine which sunbird species contributed the most to nestedness, we subtracted a second measure of nestedness (N_i_*), calculated after randomising the contributions of each species to N_tot_, according to methods described in Saavedra et al. [3] which are based on [9].

To compute the contribution of one bird species to the nestedness of the entire matrix, we calculated the difference in the nested NODF scores between the observed matrix and the average nestedness score of 1000 matrices, where the entries associated with the sunbird of interest were randomised. To test whether the measure of nestedness we obtained for the network (i.e. NODF) was significantly different from that expected by chance alone, we implemented two null models. First we randomized the matrix while preserving the number of presences (using the “r00” algorithm implemented in vegan’s null model function). Secondly we implemented the curveball algorithm in the R package ‘vegan’, which constrains connectance and species degree [43].

With the “species level” function in the bipartite package, we determined network connectance, the realised interactions (number of interaction partners) of a given species divided by the total number of possible mutualistic interactions (total number of prospective partners) (Eq 1):

 C=L/Ij (1)

Where C = connectance, L = number of realised links in a network, I = number of tree species, and J = number of sunbird species.

Individual species connectance was calculated separately for each sunbird species [40]; and species connectance was calculated as:

$$C=A_{j}/I$$ (2)

Where A_j_ = total number of interactions of species j (sunbirds) and I = number of tree species.

To determine whether species that contribute the most to nestedness are the least likely to survive perturbations, we ran a linear regression of species abundance (our proxy for rarity) against contribution to nestedness. A linear regression was also used to investigate the relationship between a species contribution to nestedness and its connectance.

## Results

**Fig 2:**
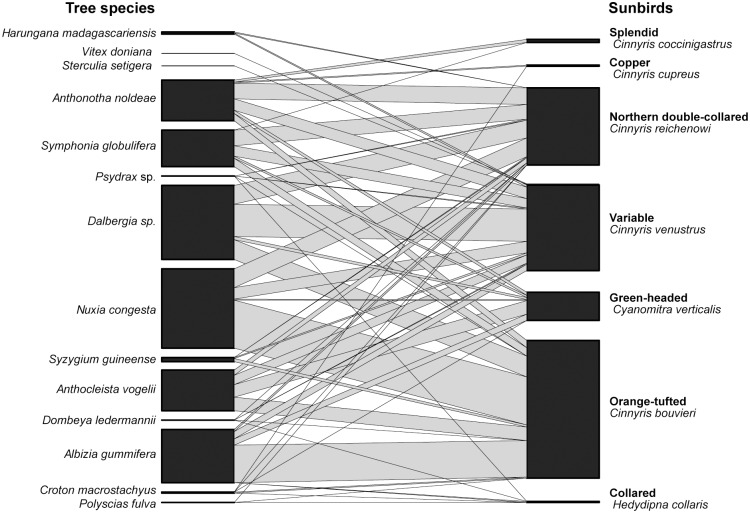
Graphical representation of the quantitative sunbird-tree pollination network at Ngel Nyaki Forest Reserve. Black rectangles represent sunbird (left) and tree (right) species. Rectangle width is proportional to the number of interactions recorded per species. Lines represent links between species; line width is proportional to the frequency of interactions (number of visits recorded by a visitor species to a plant species).

Table 1 illustrates the relative abundance of all sunbird species in our network. The least abundant sunbird the collared sunbird, *Hedydipna collaris* was not recorded at all in our transect survey while the most common species in the network, the variable sunbird, was recorded 525 times. In our network (Fig 2, S1 Table) the total number of tree species (14) was greater than the number of sunbird species (7). The overall network nestedness (NODF value) was 73.23 This degree of nestedness is substantially higher than expected if all bird-tree interactions occurred at random (P<0.005), But it is not significantly different from what would be expected from chance alone when we constrain for connectance and species’ degree using the curveball algorithm (Strona et al. 2014).

**Fig 3:**
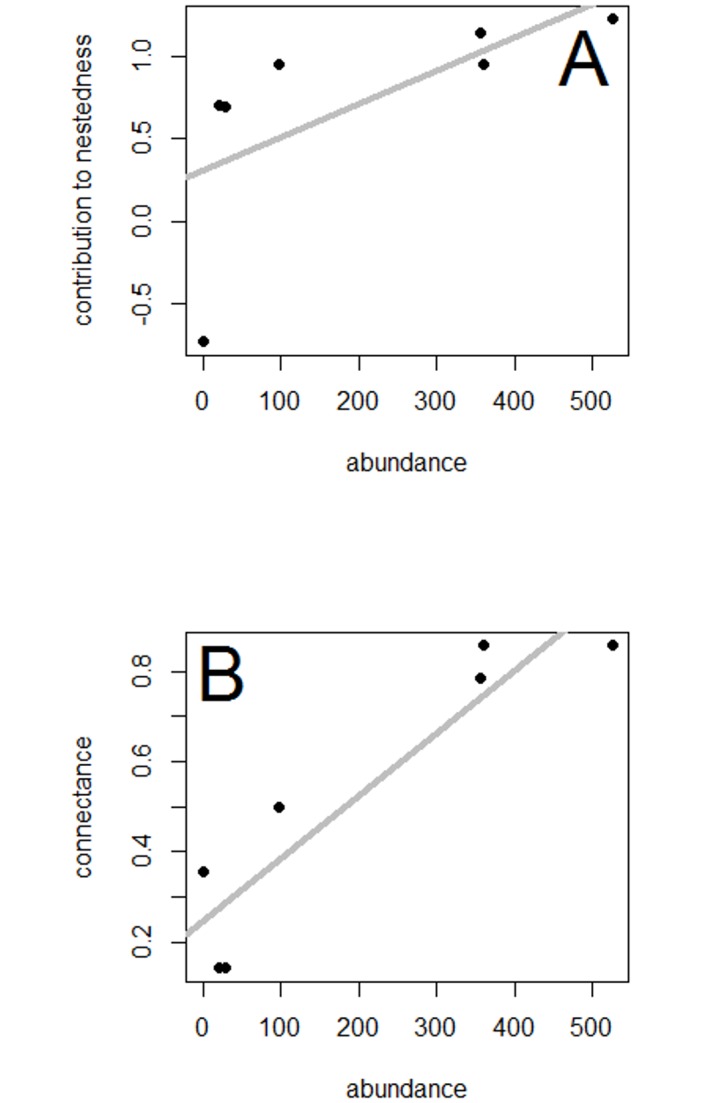
The scatterplots of the linear regressions with best fit lines of (A)- abundance vs species contribution to nestedness and (B) connectance vs species contribution to nestedness. Each black dot represents a sunbird species.

The strongest sunbird contributor to nestedness was the variable sunbird, *Cinnyris venustrus* (1.223) and the weakest contributor the Collared sunbird *C*. *collaris* (-0.729) (Table 1). The linear regression of a species contribution to nestedness against its abundance did not support the hypothesis that extinction prone species contribute more to nestedness than common ones F_1,5_ = 3.479, p = 0.1211, R^2^ = 0.2924 (Fig 3A).

The overall network connectance was 0.52, i.e. 52% of all possible interactions were realised. The two most connected sunbird species were the variable sunbird *C*. *venustrus* and *C*. *reichenowi*, both of which had a connectance value of 0.87, meaning they each interacted with 87% of the tree species in the network. The least connected sunbird species were *C*. *cupreus* and the splendid sunbird, *C*. *coccinigastrus*, both of which had a connectance value of 0.14.

We found a non-significant negative correlation between species contribution to nestedness and their connectance (coefficient = -0.584, N = 7, p = 0.16) (Fig 3B). The strongest contributor to nestedness (*C*. *venustrus*) was also one of the two equally most connected species, while the weakest contributor to nestedness (*C*. *collaris*) was ranked fourth in terms of connectance.

## Discussion

Our bird-tree visitation network is the first of its kind to be described from a West African montane habitat. While it is a sub-set of a larger network (we didn’t include insect or bat visitors in this network) it nevertheless adds to our knowledge and understanding of bird-plant visitation networks and provides valuable base-line data from which we can refine both our questions and empirical methods.

The network was unusual in that its imbalance of plant and animal species was in favour of plants [33] (14 tree vs seven sunbird species respectively). Presumably this is because we focused on birds, if we had included insect and/or bat visitors into our network the balance may well have been different. As in most mutualistic networks most links were weak and only a few were strong [33]. Three of the four strongest links in the network undoubtedly represent pollination visitations to some extent. We know from seed set exclusion experiments that *A*. *noldea* is dependent on sunbirds for pollination, and that the northern double collared sunbird is a key pollinator [32]. Moreover, despite their small flowers most obviously adapted for insect pollination, the links between the tree species *Nuxia congesta* and *Albizia gummifera* and the orange tufted sunbird may well reflect pollination because the orange tufted sunbird carried significant pollen loads of both species at NNFR [36]. Moreover African sunbirds are known to be relatively unspecialised in terms of morphological adaptations to nectar feeding [29, 30]. To what extent all links (flower visitations) in our network represent pollination rather than nectar robbing, catching visiting insects or even florivory [44, 45] is under investigation. An increase in cheating in visitation networks may destroy nestedness and enforce modularity by which the structure would slow down the spread of disturbances [17, 46].

Our first question in this study was about the resilience of the sunbird-tree pollination network; arguably nestedness provides some indication of the potential for networks to rebound from perturbations [3, 13, 15, 47] but see [6]. Our results were inconclusive; based on our first null model in which we randomized the matrix while preserving the number of presences, the degree of nestedness is substantially higher than expected if all bird-tree interactions occurred at random. However when we constrained both connectance and species degree our network was no more nested than what would be expected from random matrices, which suggests it may be more sensitive to loss of a single species than a more nested network [12]. In comparison with another study where a NODF value has been used to predict network stability [48], our network NODF value of 73.2 is less than that observed in the very robust network of solitary bees—oil flowers (NODF = 84.0) but is markedly higher than the values these authors observed in 22 whole pollination networks from the Interaction Web Database (NODF = 0.32 +/- 0.18). Just how the ecology of the sunbirds in the network influences this NODF value needs investigation. Many sunbirds feed on herbs as well as trees [30], while many of the trees in the network are likely pollinated by insects, so that there may well be considerable redundancy in the system. The only confirmed specialization is between *A*. *noldae* and sunbirds [32], and during this study *A*. *noldea* was visited and likely pollinated by six of the seven sunbird species in the network. The fact that some visitors to our network were nectar robbers [31] will affect nestedness, and possibly increase modularity [46]. Taken together these ecological observations suggest the network is likely to be relatively robust, because losing a species of either bird or tree is unlikely to collapse the network.

We found no evidence in support of our hypothesis that extinction prone species contribute the most to nestedness. Arguably the seven sunbird species in our network made more or less equivalent contributions to nestedness so that in this case their abundance and conservation extinction risk status apparently made little difference. Moreover the Variable sunbird, the most abundant sunbird species, contributed the most to nestedness, and the Orange-tufted sunbird (a common species with low extinction risk), ranked second in contribution to nestedness is further indication that the positive effect of rarity on species contribution to nestedness may not be universal. Moreover, the rarest sunbird species of all, so rare that it was never observed during the survey, contributed the least to nestedness and was the only negative contribution (Table 1). The significance of this is unclear. While it may indicate that its contribution was functionally less than all the other species with positive values, the small network and correspondingly low statistical power make this inconclusive.

Our third objective was to test whether strong contributors to nestedness have low connectance. Again, our results did not support this hypothesis. Given our small statistical power it is unclear as to the relevance of our findings, but the strongest contributor to nestedness (the Variable sunbird) was one of the two most connected species. The markedly weakest contributor to nestedness, the Collared sunbird was ranked fourth out of five connectance values (Table 1).

Despite the small network and consequent low statistical power of the results, our findings are important in that they challenge current network theory; we did not detect a significant link between a species’ extinction proneness and its contribution to nestedness [3]. Being an empirical study in a remote and vulnerable habitat, internationally recognised for its birdlife [35, 38, 49], it is important that we get it right. Integrating network theory into management decisions [28] should be useful but combining theory with field work can be challenging. Future research needs to explore why we found discrepancies between theory and empirical results. Our network is not unique in being small, open and including visitors with very different relative contributions to pollination vs eg. nectar robbing [31, 45]. Not all sunbird species are equally dependent on tree species for nectar or forests for habitat, for example Horak et al. [38] found that in Cameroon the orange tufted sunbird preferred open mountain vegetation to montane forest. Co-occurring sunbird species use different habitat niches [49, 50] and generally speaking trees tend to be less specialised to bird pollination than are herbs [1]. More statistical power could be gained if the observational data were increased by looking at larger bird/plant pollination networks, for example the New world hummingbirds. This may allow clarification of the significance of our findings. Alternatively our proxies for extinction proneness may not be accurate; most of the rare, extinction prone species in our study site are relatively abundant elsewhere in Nigeria and it is possible that this makes them less extinction prone in NNFR. This seems unlikely, given the remoteness of the Mambilla Plateau, but it cannot be ruled out. In contrast, the most common sunbird species within NNFR are scarce or absent elsewhere in West Africa. For instance, the Northern double-collared and orange- tufted sunbirds were among the most abundant species in the reserve yet they are regionally restricted to NNFR and Obudu plateau in the whole of West Africa [39]. In this study we have used a sunbird-tree visitation network. If we had used a sunbird-tree pollination network or even some intermediate proxy such as pollen load, and therefore considered factors more tightly aligned with fitness, our results may have varied. Therefore future research should focus on how nestedness and connectance interact with other determinants of network structure such as dietary preferences, phenology, and trait-matching. While difficult to do, such empirical data are necessary in order to provide even more useful insights into the various forces mediating network structure and how these forces affect the survival of species and the stability of networks.

## Supporting Information

S1 Table Flower web data. (CSV)

## References

[1] Allen-Wardell G, Bernhardt P, Bitner R, Burquez A, Buchmann SL, Cane JH, et al The potential consequences of pollinator declines on the conservation of biodiversity and stability of food crop yields. Cons. Biol. 1998; 12: 8–17.

[2] Burkle LA, Marlin JM, Knight TM. Plant-pollinator interactions over 120 years: Loss of species, co-occurrence, and function. Science. 2013; 339: 1611–1615. 10.1126/science.1232728 23449999

[3] Saavedra S, Stouffer DB, Uzzi B, Bascompte J. Strong contributors to network persistence are the most vulnerable to extinction. Nature. 2011;478(7368): 233–235. 10.1038/nature10433 21918515

[4] Vidal MM, Hasui E, Pizo MA, Tamashiro JY, Silva WR, Guimaraes PR Jr. Frugivores at higher risk of extinction are the key elements of a mutualistic network. Ecology. 2014;95(12): 3440–3447.

[5] Domínguez-García V, Munõz MA. Ranking Species in Mutualistic Networks. Sci Rep. 2015;5: 8182 10.1038/srep08182 25640575 PMC4313099

[6] James A, Pitchford JW, Plank MJ. Disentangling nestedness from models of ecological complexity. Nature. 2012;487(7406): 227–230. 10.1038/nature11214 22722863

[7] James A, Pitchford JW, Plank MJ. "Disentangling nestedness" disentangled Reply. Nature. 2013;500(7463): E2–E3. 23969465 10.1038/nature12381

[8] Saavedra S, Stouffer DB. "Disentangling nestedness" disentangled. Nature. 2013; 500(7463): E1–E2. 10.1038/nature12380 23969464

[9] Bascompte J, Jordano P, Melián CJ, Olesen JM. The Nested Assembly of Plant-Animal Mutualistic Networks. Proc Nat Acad Sci USA. 2003;100(16): 9383–9387. 10.1073/pnas.1633576100 12881488 PMC170927

[10] Allesina S, Tang S. Stability criteria for complex ecosystems. Nature. 2012;483(7388): 205–208. 10.1038/nature10832 22343894

[11] Olesen JM, Bascompte J, Elberling H, Jordano P. Temporal dynamics in a pollination network. Ecology. 2008; 89(6): 1573–1582. 18589522 10.1890/07-0451.1

[12] Tylianakis JM, Laliberté E, Nielsen A, Bascompte J. Conservation of species interaction networks. Biol Cons. 2010;143(10): 2270–2279.

[13] Thébault E, Fontaine C. Stability of ecological communities and the architecture of mutualistic and trophic networks. Science. 2010;329(5993): 853–856. 10.1126/science.1188321 20705861

[14] Dunne JA, Williams RJ, Martinez ND. Food-web structure and network theory:The role of connectance and size. Proc Nat Acad Sci USA. 99(20): 12917–12922. 10.1073/pnas.192407699 12235364 PMC130560

[15] Memmott J. The structure of a plant-pollinator food web. Ecol. Lett. 1999; 2(5): 276–280. 10.1046/j.1461-0248.1999.00087.x 33810635

[16] Armbruster S, Fenster CB, Dudash MR. Pollination “principles” revisited: specialization, pollination syndromes and the evolution of flowers. Det Norske Videnskaps-akademi. I. Matematisk Naturvidenskapelige Klasse, Skrifter, Ny Serie. 2000;39: 179–200.

[17] Olesen JM, Bascompte J, Dupont YL, Jordano P. The modularity of pollination networks. Proc Nat Acad Sci. 2007;104(50): 19891–19896. 10.1073/pnas.0706375104 18056808 PMC2148393

[18] Bewick S, Brosi BJ, Armsworth PR. Predicting the effect of competition on secondary plant extinctions in plant—pollinator networks. Oikos. 2013;122(12): 1710–1719.

[19] Kaiser-Bunbury CN, Traveset A, Hansen DM. Conservation and restoration of plant—animal mutualisms on oceanic islands. Pers Plant Ecol, Evol Syst. 2010;12(2): 131–143.

[20] Memmott J, Waser NM, Price MV. Tolerance of Pollination Networks to Species Extinctions. Proc Nat Acad Sci USA. 2004; 271(1557): 2605–2611. 10.1098/rspb.2004.2909 PMC1691904 15615687

[21] Borgella RJ, Snow AA, Gavin TA. Species richness and pollen loads of hummingbirds using forest fragments in Southern Costa Rica. Biotropica. 2001; 33: 90–109.

[22] Courter JR, Johnson RJ, Bridges WC, Hubbard KG. Assessing migration of ruby-throated hummingbirds (*Archilochus colubris*) at broad spatial and temporal scales. Auk. 2013;130: 107–117.

[23] McKinney AM, CaraDonna PJ, Inuoye DW, Barr B, Bertelsen CD, Waser NM. Asynchronous changes in phenology of migrating Broad-tailed Hummingbirds and their early-season nectar resources. Ecology. 2012;93: 1987–1993. 23094369 10.1890/12-0255.1

[24] Geerts S, Pauw A. The cost of being specialized: pollinator limitation in the endangered geophyte *Brunsvigia litoralis* (Amaryllidaceae) in the Cape Floristic Region of South Africa. S A J Bot. 2012;78:159–164.

[25] Pauw A, Laow K. Urbanization drives a reduction in functional diversity in a guild of nectar-feeding birds. Ecol Soc. 2012;17(2): 27.

[26] The IUCN Red List of Threatened Species. Version 2015–4. < www.iucnredlist.org

[27] Regan EC, Santini L, Ingwall-King L, Hoffmann M, Rondinini C, Symes A, et al Global trends in the status of bird and mammal pollinators. Conserv Lett. 2015;8(6): 397–403.

[28] Kaiser-Bunbury CN, Blüthgen N. Integrating network ecology with applied conservation: a synthesis and guide to implementation. AOB Plants. 2015; 7: plv076; 10.1093/aobpla/plv076 26162897 PMC4564002

[29] Cheke RA, Mann CF, Allen R. Sunbirds: A guide to the Sunbirds, Flowerpeckers, Spiderhunters and Sugarbirds of the World. 2001 Christopher Helm UK and Yale University Press, USA.

[30] Janeček Š, Riegert J, Sedláček O, Bartoš M, Hořák D, Reif J, et. al Food selection by avian floral visitors: an important aspect of plant—flower visitor interactions in West Africa. Biol J Linn Soc. 2012; 107(2): 355–367.

[31] Nsor CA, Chapman HM. A preliminary investigation into the avian pollinators of three tree species in a Nigerian montane forest. Malimbus. 2013;35: 38–49.

[32] Beavon M, Chapman HC. Andromonoecy and high fruit abortion in *Anthonotha noldeae* in a West African montane forest. Plant Syst Evol. 2011;296: 217.

[33] Vázquez DP, Blüthgen N, Cagnolo L, Chacoff NP. Uniting pattern and process in plant-animal mutualistic networks: a review. Oxford University Press; 2009. 10.1093/aob/mcp057 PMC2701748 19304996

[34] Viera MC, Almeida-Neto M. A simple stochastic model for complex co-extinctions in mutualistic networks: robustness decreases with connectance. Ecol Lett. 2015;18(2):144–152. 10.1111/ele.12394 25431016

[35] Chapman JD, Chapman HM. The Forests of Taraba and Adamawa States, Nigeria An Ecological Account and Plant Species Checklist. University of Canterbury Press; 2001.

[36] Nsor CA. Sunbird pollination and the fate of strong contributors to a mutualistic network in a West African Montane Forest. PhD Thesis University of Canterbury, NZ. 2015.

[37] Bibby CJ, Burgess N, Hill D, Mustoe S. Bird Census Techniques. Academic New York 2000.

[38] Hořák D, Sedláček O, Reif J, Riegert J, Pešata M. When savannah encroaches on the forest: thresholds in bird—habitat associations in the Bamenda Highlands, Cameroon. A J Ecol. 2010;48(3): 822–827.

[39] Borrow N, Demey R. A Guide to the Birds of Western Africa. Princeton University Press 2001.

[40] Dormann CF, Fründ J, Blüthgen N, Gruber B. Indices, graphs and null models: analysing bipartite ecological networks. Open Ecol J. 2009; 2:7–24.

[41] Almeida-Neto M, Ulrich W. A straightforward computational approach for measuring nestedness using quantitative matrices. Environ Modell Softw. 2008;26(2): 173–178.

[42] R Core Team. R: A language and Environment for Statistical Computing, R Foundation for statistical Computing, Vienna, Austria 2013 http://www.R-project.org/.

[43] Strona G, Nappo D, Boccacci F, Fattorini S, San-Miguel-Ayanz J. A fast and unbiased procedure to randomize ecological binary matrices with fixed row and column totals. Nat Commun. 2014;5: 4114 10.1038/ncomms5114 24916345

[44] King C, Ballantyne G, Willmer PG, Freckleton R. Why flower visitation is a poor proxy for pollination: measuring single‐visit pollen deposition,with implications for pollination networks and conservation. Method Ecol Evol. 2013; 4(9): 811–818.

[45] Padyšáková E, Bartoš M, Tropek R, Janeček Š. Generalization versus specialization in pollination systems:visitors, thieves, and pollinators of *Hypoestes aristata* (Acanthaceae). PloS One. 2013; 8(4): e59299 10.1371/journal.pone.0059299 23593135 PMC3622670

[46] Genini J, Morellato LPC, Guimarães PR, Olesen JH. Cheaters in mutualism networks. Biol Lett. 2010; 6:494–497. 10.1098/rsbl.2009.1021 20089538 PMC2936203

[47] Bastolla U, Fortuna MA, Pascual-García A, Ferrera A, Luque B, Bascompte J. The architecture of mutualistic networks minimizes competition and increases biodiversity. Nature.2009;458: 1018–1020. 10.1038/nature07950 19396144

[48] Bezzerra EL, Machado IS, Mello MA. Pollination networks of oil-flowers: a tiny world within the smallest of all worlds. J A Ecol. 2009;78: 1096–1101. 10.1111/j.1365-2656.2009.01567.x 19515098

[49] Ezealor A. Nigeria: Important Bird Areas in Africa and associated islands:priority sites for Conservation. BirdLife Cons series 2001;11: 673–682.

[50] Geerts S, Pauw A. Hyper-specialization for long-billed bird pollination in a guild of South African plants: the Malachite Sunbird pollination syndrome. S A J Ecol. 2009;75: 699–706.

